# Performance Analysis of State-of-the-Art CNN Architectures for LUNA16

**DOI:** 10.3390/s22124426

**Published:** 2022-06-11

**Authors:** Iftikhar Naseer, Sheeraz Akram, Tehreem Masood, Arfan Jaffar, Muhammad Adnan Khan, Amir Mosavi

**Affiliations:** 1 Faculty of Computer Science & Information Technology, The Superior University, Lahore 54600, Pakistan; iftikharnaseer@gmail.com (I.N.); sheeraz@superior.edu.pk (S.A.); tehreem.masood@superior.edu.pk (T.M.); arfan.jaffar@superior.edu.pk (A.J.); 2 Department of Software, Gachon University, Seongnam 13120, Korea; 3 John von Neumann Faculty of Informatics, Obuda University, 1034 Budapest, Hungary; mosavi.amirhosein@uni-nke.hu; 4 Institute of Information Engineering, Automation and Mathematics, Slovak University of Technology in Bratislava, 81107 Bratislava, Slovakia; 5 Faculty of Civil Engineering, Technical University of Dresden, 01062 Dresden, Germany

**Keywords:** LeNet, AlexNet, deep learning, LUNA16, machine learning, artificial intelligence, cancer research, lung cancer, medical image analysis, big data

## Abstract

The convolutional neural network (CNN) has become a powerful tool in machine learning (ML) that is used to solve complex problems such as image recognition, natural language processing, and video analysis. Notably, the idea of exploring convolutional neural network architecture has gained substantial attention as well as popularity. This study focuses on the intrinsic various CNN architectures: LeNet, AlexNet, VGG16, ResNet-50, and Inception-V1, which have been scrutinized and compared with each other for the detection of lung cancer using publicly available LUNA16 datasets. Furthermore, multiple performance optimizers: root mean square propagation (RMSProp), adaptive moment estimation (Adam), and stochastic gradient descent (SGD), were applied for this comparative study. The performances of the three CNN architectures were measured for accuracy, specificity, sensitivity, positive predictive value, false omission rate, negative predictive value, and F1 score. The experimental results showed that the CNN AlexNet architecture with the SGD optimizer achieved the highest validation accuracy for CT lung cancer with an accuracy of 97.42%, misclassification rate of 2.58%, 97.58% sensitivity, 97.25% specificity, 97.58% positive predictive value, 97.25% negative predictive value, false omission rate of 2.75%, and F1 score of 97.58%. AlexNet with the SGD optimizer was the best and outperformed compared to the other state-of-the-art CNN architectures.

## 1. Introduction

Artificial intelligence (AI) has proven to be a significant success in every field of life [1]. Artificial intelligence is the mimic of human intelligence used by computer programs. It has a subset named the machine learning (ML) technique that helps to train algorithms in making decisions [2]. Currently, deep learning technology has become a promising approach for clinical detection systems [3]. A deep learning computer-aided diagnosis system has been used to analyze medical images, which has proven to be a remarkable advancement in various medical applications [4]. Deep learning algorithms have the potential to solve real-world complex problems, especially in image analysis and computer vision [5]. Convolutional neural network (CNN) is a deep learning technique used in image and text recognition [6]. CNN has proven to have remarkable performance in understanding image segmentation, image classification problems, and detection [7]. Medical imaging is a core standard for the early diagnosis, detection, and treatment of several diseases. Medical images are kept in Digital Imaging and Communications in Medicine (DICOM) format, which can be quickly accessed for quantitative and qualitative analysis [8]. 

Medical imaging modalities comprise multidisciplinary techniques to attain an accurate diagnosis of various diseases. There are various medical image modalities such as computed tomography, X-ray angiography, mammography, digital radiography, radio fluoroscopy, and computed radiography that are used to analyze image processing [9]. Computed tomography (CT) images are an easier and more accurate method for the correct detection of various diseases [10]. However, it is tedious for medical experts to manually analyze CT scans without any error [11]. In the last few years, a low-dose CT scan has been used to screen for lung cancer diseases that has led to a decrease in the mortality rate of lung cancer [12]. 

The authors of [13] presented a framework based on deep learning to detect lung cancer and pneumonia abnormalities. The deep learning technique, named Modified AlexNet (MAN), was presented to detect two classes, normal and pneumonia class, by using X-ray images. The MAN technique was applied to classify lung cancer by using a support vector machine (SVM) model. The MAN method performance was evaluated with other deep learning approaches such as ResNet50, VGG19, VGG16, and AlexNet and achieved 97.27% accuracy by using the LIDC-IDRI dataset. Another study [14] presented a network based on a deep convolutional neural network, named AlexNet. The network consisted of an eight-layer two-architecture deep CNN to classify malignant and benign nodules. The network extracted the features automatically from CT scan images. Binary cross-entropy was applied to improve precision in the training phase as well as validation accuracy. The lung nodule classification model achieved 99% training precision and 97% validation accuracy. Similarly, the authors of [15] introduced a lung cancer detection method based on a deep learning algorithm using the segmentation method. Five-fold cross-validation was applied to train and validate the deep learning-based model. In another study, the authors of [16] presented a CAD system for pulmonary pure ground-glass nodules based on the convolutional neural network. In line with the above, various research works have been presented where diverse optimization algorithms have been applied to achieve an improvement in performance for the detection of lung cancer diseases. In this research work, we have investigated and explored an optimization algorithm based on a convolutional neural network to detect lung cancer. Furthermore, this work exploited the optimizers RMSprop, Adam, and SGD as well as their implementation with CNN architectures. 

The remaining part of the paper has been organized as follows: Section 2 discusses prior works published in recent years. Section 3 introduces the materials and methods based on CNN, and Section 4 demonstrates the experimental results. Section 5 discusses the present, and finally, limitations and future works are discussed in Section 6. 

## 2. Related Work

Convolutional neural networks have been implemented to solve various visual problems since the late 1980s. LeCun et al. used a first-time backpropagation algorithm in multilayered CNN, namely ConvNet, to recognize handwritten zip codes in 1989 [14]. Khehrah et al. presented a pulmonary nodule detection system using shape-based and statistical features in CT images [17]. Another study [18] introduced lung nodule detection based on an artificial neural network using texture and shape features. The model achieved an accuracy of 89.62%. Similarly, the authors of [19] proposed a lung cancer detection model based on ANN that achieved 96.67% accuracy. Miah et al. [20] presented a lung cancer detection system using a neural network from CT images that obtained 96.67% accuracy. LeCun et al. [21] suggested another advanced version of ConvNet called LeNet-5 to classify characters in a document in 1998.

In [22], the researchers applied the LeNet-5 model to classify benign and malignant pulmonary nodules in thoracic CT images. Lung Image Database Consortium and Image Database Resource Initiative (LIDC-IDRI) datasets were obtained for the experimental results. The 10-folder cross-validation was implemented to evaluate the model classification. The LeNet-5 achieved 97.041% accuracy in classifying benign and malignant nodules and 96.685% accuracy in classifying mild malignancies and serious malignancies. Another study [23] presented a hybrid version of a convolutional neural network for the classification of pulmonary nodules based on LeNet and AlexNet. The hybrid framework used LeNet’s layers and parameters of AlexNet. A total number of 1018 CT images was obtained from the LIDC-IDRI dataset to train and evaluate the agile convolutional neural network. Various parameters such as kernel size, batch size, learning rate, and weight initialization played an important role in achieving high accuracy. The framework achieved 0.822 accuracy and 0.877 area under the curve, with the kernel size set to 7 × 7, the batch size at 32, and the learning rate at 0.005. Gaussian and dropout were also applied in this work.

Krizhevsky et al. [24], introduced the first deep convolutional neural network named AlexNet in 2012. AlexNet outperformed in the ImageNet Large Scale Visual Recognition Challenge (ILSVRC)-2012 and proved to be a pervasive breakthrough in the performance of CNN.

Agarwal et al. [25] investigated a framework to detect and classify lung cancer based on AlexNet CNN. In the first step, the green channel extracted was from the original color CT image. Multilevel thresholding was used to extract lung regions. The morphological and thresholding segmentation methods were applied to separate non-affected and affected regions. After segmentation of the tumor regions, AlexNet-CNN was classified into benign and malignant with 96% accuracy.

In [26], the researchers recommended two architectures named straight 3D-CNN and hybrid 3D-CNN for the classification of pulmonary nodules. The features extraction method was the same in both models, but the classifiers differed. The model 3D-CNN used a softmax classifier to classify the pulmonary CT images, and the hybrid 3D-CNN used the radial basis function (RBF)-based support vector machine (SVM) classifier for classification purposes. The experimental results indicated that the hybrid 3D-CNN and straight 3D-CNN models achieved better accuracy. Nevertheless, the approach obtained an accuracy of 91.8%, specificity of 94.23%, sensitivity of 88.53%, and precision of 91.9% as compared to the 3D-CNN model with a softmax classifier.

Rao et al. [27] exploited the classification of lung tumors by using convolutional neural networks. The suggested CanNet approach consisted of two convolution layers, a pooling layer, a dropout layer, and a final fully connected layer. Lung Image Database Consortium (LIDC) was used to train and evaluate an artificial neural network, LeNet, and CanNet networks. The dataset comprised 1018 patients’ CT scan data, and each CT scan consisted of almost 150 to 550 DICOM images. Artificial neural networks, LeNet, and CanNet achieved accuracies of 72.50%, 56.0%, and 76.00%, respectively. The CanNet model outperformed compared to the ANN and LeNet networks.

Lin et al. [28] presented a lung nodule classification model comprising a Taguchi-based convolutional neural network. Useful information obtained with fewer experiments is the most significant advantage of the Taguchi technique. A total number of 245,931 images including CT scans and X-ray images were obtained to evaluate the performance of the AlexNet model. The experimental results demonstrated that AlexNet with the Taguchi-based model used less training time as compared to other approaches.

The authors of [29] introduced a computer-aided scheme based on a convolutional neural network with AlexNet architecture to diagnose and classify lung cancer. The lung cancer CT scan dataset was collected from Iraqi Hospitals that were used to train and test the system. For training purposes, 70% was used to train and 30% was used for testing. The dataset was classified into three categories: normal, benign, and malignant, and consisted of 110 CT scans. The system achieved 93.548% accuracy, 97.102% precision, 95% specificity, 95.714% sensitivity, and 96.403% F1 score. The authors of [30] investigated the effects of Visual Geometry Group 16 (VGG16) and Visual Geometry Group 19 (VGG19) on ImageNet Challenge 2014. VGG16 and VGG19 consisted of 16 and 19 weight layers, respectively. The input size comprised 224 × 224 RGB images, and a convolutional filter size of 3 × 3 was used in both networks, providing significant improvements in the image recognition process. The experimental results exploited that the depth representation is beneficial to classifying problems and increased state-of-the-art accuracy. Both networks were also tested on other datasets and achieved high accuracy as compared to other techniques.

Another study [31] introduced a technique to detect early lung cancer by using a deep learning genetic algorithm. In the preprocessing method, three techniques were applied: the histogram stretching technique was used to enhance the contrast of the raw image; the Wiener filter was used to remove noise; the image was cropped into 224 × 224 for VGG16 and 227 × 227 for VGG19 for the AlexNet architecture. Low-dose computed tomography (LDCT) images were used, and three CNN architectures, VGG16, VGG19, and AlexNet, were applied to extract features. A genetic algorithm was applied to select the most relevant features. Finally, K-nearest neighbor (KNN), decision tree, and SVM classifiers were investigated to classify pulmonary lung nodules. The experimental results indicated that VGG19 with a support vector machine classifier obtained a remarkable 96.3% accuracy, sensitivity of 97.5%, and specificity of 95% as compared to other CNN models and classifiers.

The authors of [32] presented an accurate lung segmentation technique based on VGG-16 and dilated convolution network. Dilated convolution used a dilation rate parameter and indicated an expansion of the size of the respective field. The hypercolumn features technique was used to fuse multi-scale convolution features to enhance the robustness of the lung segmentation technique. The modified VGG16 was used, followed by the multilayer perceptron (MLP) and ReLU activation function. The method achieved a dice similarity coefficient of 0.9867.

Another study based on VGG16 [33] introduced VGG16 with a boosting technique for the identification of the pathological types of lung cancer. The dataset consisted of 125 patients with early-stage lung cancer and was enhanced by using reproducing, shifting, and revolving operations. VGG16-T comprised five convolution layers with a kernel size of 3 × 3. It was found that the boosting strategy enhanced the accuracy, and three weak classifiers can be adequate enough to make a strong classifier. Finally, the softmax function was applied to identify the pathological type of lung cancer by using CT images. The experimental results of VGG16-T with boosting achieved 85% accuracy, which was better than the other techniques, ResNet-34, DenseNet, and AlexNet.

Similarly, the authors of [34] presented a lung cancer detection system by using a transfer learning technique. The suggested method reduced the processing time by using a maximum dropout ratio, and it decreased overfitting in the learning phase. GoogleNet, AlexNet, and ResNet50 transfer learning convolutional neural network architectures were applied to detect lung cancer. LIDC, a publicly available dataset, was used to train and test the pre-trained model as well as the suggested model and achieved high accuracy as compared to pre-trained methods.

Another study [35] based on ResNet architecture presented a method for pulmonary nodule classification. The proposed model was based on 18 layers of ResNet and achieved 89.90% accuracy using LIDC-IDRI. In [36], an inception module CNN classifier achieved 88.67% validation accuracy for the detection of pulmonary nodules on the AIA-INF publicly available dataset. Similarly, in [37], the Darknet-53 CNN-based architecture was applied for pulmonary nodule detection and achieved 70.5% to 73.9% accuracy on the LUNA16 dataset. Many other studies covered other cancer types such as thyroid, breast, skin, colon, and blood cancers. In [38], the Xception neural network was applied to thyroid cancer for the early detection of malignant nodules. The framework adopted three-level multi-channel and real-world datasets, which were used to evaluate the proposed approach. Another cancer type, colon carcinoma [39], was adopted to be used in a convolutional neural network for classification task. A study [40] conducted on the early detection of breast cancer was based on fused and deep learning approaches; similarly, another study [41], empowered with a deep learning technique, exploited breast cancer and its stages, such as mucinous carcinoma, papillary carcinoma, ductal carcinoma, and lobular carcinoma. A comprehensive study [42] was investigated for the automatic detection of skin lesions based on various CNN architectures. Similarly, another framework [43] based on an optimal CNN exploited the automatic detection of skin cancer. The proposed framework comprised an advanced version of the whale optimization algorithm, and the results were analyzed with 10 different techniques.

A summary of previous related work is mentioned in Table 1. The limitations of previous studies are mentioned, such as deep knowledge [17,18,19,20], which is required to obtain handcrafted features. The studies [25,29,31] were based on lesser amounts of images and on imbalanced datasets. The research works [22,23,26] focused on hybrid techniques that created complexity of the model, while different architectures were used in some research works [35,36,37] to improve accuracy.

The following are this work’s key contributions:

(a) The main contribution of this study is to provide a performance-oriented analysis by combining deep learning algorithms with different optimizers for the classification of lung cancer.
(b) We have implemented CNN architectures with Adam, SGD, and RMSprop optimizers on the LUNA16 publicly available dataset.
(c) It was observed that the AlexNet architecture with the SGD optimizer achieved the best results on the LUNA16 dataset.
(d) Finally, AlexNet with the SGD optimizer approach achieved the highest accuracy as compared with other existing techniques for lung cancer classification.

## 3. Materials and Methods

**Figure 1:**
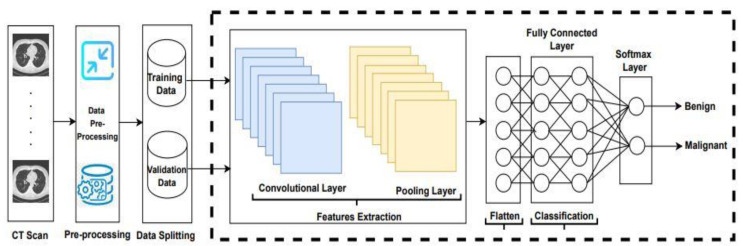
Convolutional neural network architecture.

Lung cancer has become the main reason for cancer deaths all over the world, as its symptoms appear late. Consequently, a significant detection system is required to detect lung cancer in patients at its early stages. In this study, the existing algorithms LeNet, AlexNet, VGG16, ResNet-50, and Inception-V1, with the Adam, SGD, and RMSprop optimizers were applied to classify lung cancer, and an example is shown in Figure 1.

The overall procedure adopted to apply convolutional neural network architectures is demonstrated in Figure 1. In the first step, the LUng Nodule Analysis 2016 (LUNA-16) dataset obtains from the publicly available lung cancer nodules [44]. The LIDC-IDRI database is publicly available from The Cancer Imaging Archive (TCIA). This database contains a total number of 1018 CT scans. CT scan images are associated with XML files annotated by four experienced radiologists. Thin-slice CT scans play a significant role in the detection of pulmonary nodules; therefore, the authors of [45] discarded slices that were greater than 3 mm thick, missing slices, and those with inconsistent slice spacing. A total number of 888 scans provided as MetaImage (.mhd) images are accessible from the LUNA-16 website. Two methods are applied on LUNA16 for training and validation purposes. In the first method, a dataset is randomly split into 80% and 20%, and the second method is based on 5-fold cross-validation. After splitting and 5-fold cross-validation, the dataset is forwarded to the CNN architectures to extract the features.

CNNs have various parameters and hyperparameters such as neurons, number of layers, weights, biases, stride, filter size, activation function, learning rate, etc. Convolution operation plays a significant role in image feature extraction [46]. Two types of filters, large size filters and small size filters, are used to extract various information. Large-sized filters are used to extract coarse-grained information, and small-sized filters are used to extract fine-grained information.

LeNet was developed by LeCun in 1998 for zip code recognition. In LeNet, a convolve filter size of 5 × 5 with a stride of 1 was used, and in the subsampling (pooling) layer, a filter size of 2 × 2 was applied with a stride of 2. AlexNet was the first CNN-based method that won the ImageNet Scale Visual Recognition Challenge in 2012. AlexNet comprises 5 convolutions, 3 pooling, and 3 fully connected (FC) layers. The input image size was 227 × 227 × 3, and the rectified linear unit (ReLU) was applied for the first time in AlexNet. The visual geometric group (VGG) has two versions: VGG16 with 16 layers and VGG19 with 19 layers. In VGG16 and VGG19, the number of layers increases, but the size of the filters decreases. In this study, we applied LeNet, AlexNet, VGG16, ResNet-50, and Inception-V1 to classify lung cancer on the LUNA16 dataset.

Optimizers are methods or algorithms applied to minimize a loss function and to maximize the efficiency of the model. Optimizers are mathematical functions that are based on the model’s learnable parameters, and they assist to reduce the losses with updated learning rates and weights of the neural network. The learning rate (LR) is known as a tuning parameter that works in an optimization algorithm. LR determines the step size in an optimization algorithm at each iteration when moving toward finding a minimum of the loss function.

Root mean square propagation [47] is also an adaptive learning method to resolve destructive learning rates. RMSprop determines the learning rate after each iteration by using an exponentially weighted average [41].

(1) $$q_{t}=q_{t-1\;}+\left(1-ϒ\right)\times p_{t}^{2}$$

(2) $$\Delta w_{t}=-\frac{q_{t}}{\sqrt{q_{t}+\varepsilon }}\times p_{t}\;$$

(3) $$w_{t+1}=w_{t}+\eta \times \Delta w_{t}\;$$

where

η: initial learning rate;

$q_{t}$: exponential average of gradients along *w_j_*;

$p_{t}$: gradient at time t along *w_j_*;

$x_{t}$: exponential average of squares of gradients along *w_j_*;

ϒ: hyperparameter.

The adaptive moment estimation (Adam) method computes adaptive learning rates for every parameter at each iteration. It is easy to implement with less memory requirements, and it is computationally efficient. Adam uses a combination of RMSprop and gradient descent with momentum to determine the parameter values [41].

(4) $$q_{t}={ϒ}_{1}*q_{t-1}-\left(1-{ϒ}_{1}\right)\times p_{t}$$

(5) $$s_{t}={ϒ}_{2}*s_{t-1}-\left(1-{ϒ}_{2}\right)\times p_{t}$$

(6) $$\Delta w_{t}=-p_{t}\frac{q_{t}}{\sqrt{x_{t}+\varepsilon }}\times p_{t}$$

(7) $$w_{t+1}=w_{t}+\eta \times \Delta w_{t}$$

where

Ƞ: initial learning rate;

$q_{t}$: exponential average of gradients along *w_j_*;

$p_{t}$: gradient at time t along *w_j_*;

$x_{t}$: exponential average of squares of gradients along *w_j_*;

${ϒ}_{1}$ and ${ϒ}_{2}$: hyperparameters.

A stochastic gradient descent (SGD) focuses on performing updates to the model parameters one at a time; therefore, it is much faster. After each iteration, the cost function minimizes, and SGD performs frequent updates of the model parameter that causes the cost function to fluctuate heavily, which leads the gradient to jump to the global minimum. It requires less memory and permits the use of enormous datasets [41].

(8) $$f\left(x\right)=\frac{1}{n}\times \overset{n}{\underset{i=0}{\sum }}f_{i}\left(x\right)\;$$

(9) $$▽f\left(x\right)=\frac{1}{n}\overset{n}{\underset{i=0}{\sum }}▽f_{i}\left(x\right)\;$$

(10) $$x\;\leftarrow \;x-\eta ▽f_{i}\;\left(x\right)\;$$

(11) $$E_{i}▽f\left(x\right)=\frac{1}{n}\overset{n}{\underset{i=0}{\sum }}▽f_{i}\left(x\right)=▽f\left(x\right)\;$$

where

η: initial learning rate;

*x*: parameters;

*n*: training dataset;

$f_{i}\left(x\right):$ loss function;

$▽f_{i}\;\left(x\right):$ stochastic gradient.

In this study, CNN architectures with LeNet, AlexNet, VGG16, ResNet-50, Inception-V1 with the Adam, RMSprop, and SGD optimizers were applied to extract features from the LUNA16 dataset. Subsequently, the flattened layer converts the matrix to vector form and is fed to the fully connected layer for classification purposes. Finally, the classifier softmax classifies lung cancer into benign and malignant.

## 4. Results and Discussion

In this study, the comparison between state-of-the-art CNN architectures LeNet, AlexNet, VGG16, ResNet-50, and Inception-V1 for the detection of lung cancer is explained in detail by using the different optimizers, Adam, RMSprop, and SGD. The performances of the LeNet, AlexNet, VGG16, ResNet-50, and Inception-V1 architectures were validated and evaluated in terms of accuracy. These deep learning networks use binary cross-entropy loss. In this section, the results presented were achieved by LeNet, AlexNet, VGG16, ResNet-50, and Inception-V1 detection algorithms.

In this study, the performance analysis was implemented in the Keras tool using Python 3.8. The Adam, SGD, and RMSprop optimizers were employed with a learning rate of 0.001, batch size of 20, and 200 epoch values. To measure the performance of LeNet, AlexNet, VGG16, ResNet-50, and Inception-V1, various optimizers such as RMSprop, Adam, and SGD were applied, and various statistical parameters were measured to detect lung cancer.

Various statistical parameters such as accuracy [48], sensitivity [49], specificity [50], positive predictive value [51], negative predictive value [52], false omission rate [53], and F1 score were applied to evaluate the performance of the convolutional neural network architectures with the optimizers.

(12) $$Accuracy=\frac{\left(TN+TP\right)}{\left(TN+FN+FP+TP\right)}\times 100\%\;$$

(13) $$Sensitivity=\frac{TP}{\left(TP+FN\right)}\times 100\%\;$$

(14) $$Specificity=\frac{TN}{\left(TN+FP\right)}\times 100\%\;$$

(15) $$Positive\;predictive\;value\;(PPV)=\frac{TP}{\left(TP+FP\right)}\times 100\%\;$$

(16) $$Negative\;predictive\;value\;(NPV)=\frac{TP}{\left(TP+FP\right)}\times 100\%\;$$

The validation confusion matrix of the LeNet architecture is shown in Table 2, and the validation performance factors of the LeNet model with different optimizers are shown in Table 3. LeNet with the SGD optimizer achieved a 95.92% accuracy, which is higher than the RMSprop and Adam optimizers. The other statistical parameters of LeNet with the SGD optimizer such as accuracy, sensitivity, specificity, PPV, NPV, FOR, and F1-score were 95.92%, 94.76%, 97.25%, 97.51%, 94.22%, 5.78%, and 96.11%, respectively.

The validation confusion matrix of AlexNet is shown in Table 4, and the validation performance of the AlexNet model with different optimizers is shown in Table 5. AlexNet with the SGD optimizer achieved 97.42% accuracy, which was higher than the RMSprop and Adam optimizers. The other statistical parameters of AlexNet with the SGD optimizer such as sensitivity, specificity, PPV, NPV, FOR, and F1-score were 97.58%, 97.25%, 97.58%, 97.25%, 2.75%, and 97.58%, respectively.

The VGG16 validation confusion matrix is shown in Table 6, and the validation performance of the VGG16 model with different optimizers is shown in Table 7. VGG16 with the SGD optimizer achieved 93.56% accuracy, which was higher than the RMSprop and Adam optimizers. The other statistical parameters of VGG16 with the SGD optimizer such as sensitivity, specificity, PPV, NPV, FOR, and F1-score were 91.53%, 95.87%, 96.19%, 90.87%, 9.13%, and 93.80%, respectively.

The ResNet 50 validation confusion matrix is shown in Table 8, and the validation performance of the ResNet 50 model with different optimizers is shown in Table 9. ResNet 50 with the SGD optimizer achieved 96.35% accuracy, which was higher than the RMSprop and Adam optimizers. The other statistical parameters of ResNet 50 with the SGD optimizer such as sensitivity, specificity, PPV, NPV, FOR, and F1-score were 91.53%, 95.87%, 96.19%, 90.87%, 9.13%, and 93.80%, respectively.

The Inception-V1 validation confusion matrix is shown in Table 10, and the validation performance of the Inception-V1 model with different optimizers is shown in Table 11. Inception-V1 with the SGD optimizer achieved 93.56% accuracy, which was higher than the RMSprop and Adam optimizers. The other statistical parameters of Inception-V1 with the SGD optimizer such as sensitivity, specificity, PPV, NPV, FOR, and F1-score were 91.53%, 95.87%, 96.19%, 90.87%, 9.13%, and 93.80%, respectively.

Table 12 demonstrates the results obtained from AlexNet with the SGD optimizer on the Luna16 dataset. The original image was benign, and the CNN architecture AlexNet with the SGD optimizer detected the image as benign. Next, the image was benign and AlexNet with the SGD detected the image as malignant, which was wrongly predict by AlexNet. The next image was malignant, and AlexNet with the SGD optimizer detected it as benign. Finally, the actual image was malignant, and AlexNet with the SGD optimizer detected it as malignant.

**Figure 2:**
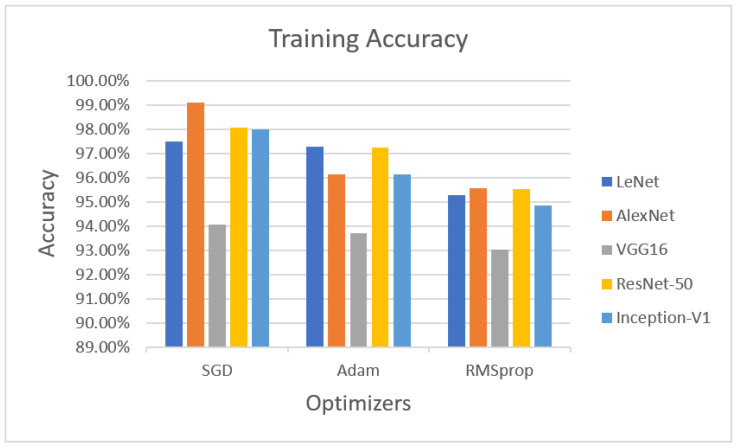
Performance evaluation with statistical parameters for LeNet, AlexNet, VGG16, ResNet-50, and Inception-V1 (training accuracy).

In the training phase, the accuracies of LeNet, AlexNet, VGG16 ResNet-50 and Inception-V1 with the optimizers are illustrated in Figure 2. LeNet with the SGD optimizer achieved 97.47% accuracy, whereas AlexNet with the SGD optimizer obtained 99.09% accuracy. VGG16 with the SGD optimizer achieved 94.07% accuracy. ResNet-50 with the SGD optimizer obtained 98.05% accuracy, while Inception-V1 with the SGD optimizer achieved 97.99% accuracy.

The next optimizer, Adam, was performed on LeNet, AlexNet, VGG16, ResNet-50, and Inception-V1. LeNet with the Adam optimizer achieved 97.26% accuracy, whereas AlexNet with Adam obtained 96.13% accuracy, VGG16 with Adam achieved 93.71% accuracy, ResNet-50 with Adam achieved 97.24% accuracy, and Inception-V1 with Adam achieved 96.12% accuracy.

The last optimizer, RMSprop, also measured for training purposes. LeNet with RMSprop achieved 95.27%, AlexNet with RMSprop obtained 95.54% accuracy, VGG16 with RMSprop achieved 93.01% accuracy, ResNet-50 obtained 95.53% accuracy, and Inception-V1 achieved 94.86% accuracy using the RMSprop optimizer.

**Figure 3:**
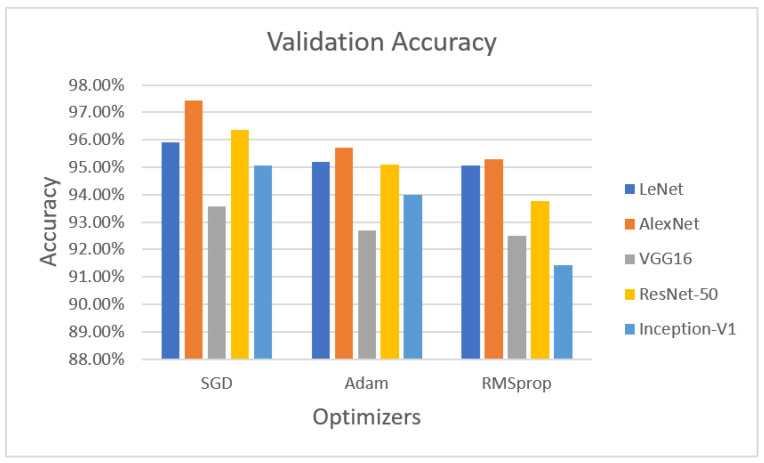
Performance evaluation with statistical parameters for LeNet, AlexNet, VGG16, ResNet-50, and Inception-V1 (validation accuracy).

Figure 3 demonstrates the validation phase, the LeNet algorithm with the SGD optimizer achieved 95.92% validation accuracy. AlexNet with the SGD optimizer obtained 97.42% accuracy, VGG16 with the SGD optimizer obtained 93.56% validated accuracy, ResNet-50 with the SGD optimizer achieved 96.35% validation accuracy, and Inception-V1 with the SGD optimizer obtained 95.06% accuracy.

The next optimizer, Adam, was performed on LeNet, AlexNet, and VGG16. LeNet with the Adam optimizer achieved 95.18% accuracy, whereas AlexNet with Adam obtained 95.71% accuracy, VGG16 achieved 92.70% accuracy, ResNet-50 with the Adam optimizer achieved 95.09% validation accuracy, and Inception-V1 with Adam obtained 93.99% accuracy.

The last optimizer RMSprop also measured for validation purposes. LeNet with RMSprop achieved 95.06%, AlexNet with RMSprop obtained 95.28% accuracy, VGG16 with RMSprop achieved 92.49% accuracy, ResNet-50 with the RMSprop optimizer achieved 93.78% accuracy, and Inception-V1 with RMSprop achieved 91.42% validation accuracy on theLUNA16 dataset.

The second method, five-fold cross-validation, was adopted to train and validate the CNN architectures with the SGD optimizer. The validation statistical analysis is shown in Table 13. When the five-fold cross-validation approach was applied, the AlexNet–SGD optimizer achieved 95.73%, 95.20%, 96.33%, 96.75%, 94.59%, 5.41% and 95.97% accuracy, sensitivity, specificity, PPV, NPV, FOR, and F1-score, respectively, which were the highest scores compared with the other CNN architectures.

The CNN-based architectures LeNet, AlexNet, VGG16, ResNet-50, and Inception-V1, with different optimizers, were evaluated in this study. It was found that AlexNet with the SGD optimizer achieved the highest accuracy of 97.42%. Table 14 presents the performance analysis of AlexNet with the SGD technique with different methods. Comparatively, the accuracy of AlexNet with the SGD was higher than the other state-of-the-art approaches. Various existing publications used different methods on the different datasets, including LIDC-IDRI, LUNA16, IQ-OTH/NCCD, ELCAP, and private lung datasets for the detection of lung cancer. AlexNet with SGD and with the five-fold cross-validation method obtained 95.73% accuracy and a 4.27% misclassification rate. AlexNet with the SGD achieved the highest accuracy of 97.42% and 2.58% of misclassification rate.

## 5. Conclusions

In recent years, lung cancer has become a dangerous disease with a low survival rate. Early diagnosis and proper treatment can increase the survival rate. In this study, various deep learning-based architectures were presented to classify lung cancer into benign and malignant. The advanced CNN architectures LeNet, AlexNet, VGG16, ResNet-50, and Inception-V1 were applied for the detection of lung cancer to analyze the performance. Various optimizers, including RMSprop, Adam, and SGD, were used to tune the CNN architectures, which provided different results. The experimental results show that the AlexNet architecture with the SGD optimizer achieved the highest validation accuracy of 97.42%, with a misclassification rate of 2.58% for the detection of lung cancer as benign or malignant while applying the five-fold cross-validation method. AlexNet–SGD achieved 95.73% accuracy, while AlexNet with the SGD optimizer outperformed compared to other state-of-the-art existing CNN architectures and optimizers.

## 6. Limitations and Future Work

The performance analysis of the state-of-the-art CNN architectures is presented in this study to classify lung cancer into benign and malignant. The study comprised five CNN architectures: LeNet, AlexNet, VGG16, ResNet-50, and Inception-V1, with the Adam, RMSprop, and SGD optimizers. Future work should include a performance analysis that can be increased to improve the classification system using other state-of-the-art CNN architectures, such as Darknet, EfficientNet, VGG19, Xception, Inception-V3, Inception-V4, Inception-ResNet-V2, and ResNeXt50. Various optimizers and cross-validation techniques can be adapted to remove the randomness effect to achieve better accuracy.

## Figures and Tables

**Table 1:** Literature survey on computational intelligence-based lung cancer detection methods.

Publications	Method	Dataset	Accuracy%	Weakness
Khehrah et al. [17]	ANN	LIDC-IDRI	92.0%	Requires handcrafted features
Xie et al. [18]	ANN	LIDC-IDRI	89.62%	Requires handcrafted features
Naseer et al. [19]	ANN	Private Lung Dataset	96.67%	Requires handcrafted features
[20]	ANN	Private Lung Dataset	96.67%	Requires handcrafted features
S. Zhang et al. [22]	LeNet-5 10 fold Cross-Validation	LIDC-IDRI	97.04%	Complexity required
Zhao et al. [23]	hybrid CNN of LeNet and AlexNet is	LIDC-IDRI	87.7%	Complexity required
Agarwal et al. [25]	AlexNet CNN	Private Lung Dataset	96.0%	Less number images
Polat et al. [26]	Hybrid 3D-CNN RBF-based	LUNA16 Lungs Data Science Bowl	91.81%	Complexity required
Al-Yasriy et al. [29]	AlexNet CNN	(IQ-OTH/NCCD) lung cancer dataset	93.548%	Use of imbalance dataset
A. Elnakib et al. [31]	VGG19 architecture and SVM classifier	Early Lung Cancer Action Project (ELCAP) database	96.25%	Less number images
Nibali et al. [35]	ResNet-18 architecture	LIDC-IDRI	89.90%	Needs to improve accuracy
Zheng et al. [36]	Inception CNN classifier	AIA-INF	88.67%	Needs to improve accuracy
Haibo et al. [27]	DarkNet-53 CNN architecture	LUNA16	73.9%	Needs to improve accuracy

**Table 2:** LeNet confusion matrix (validation).

CNN Architecture	Optimizer	True Negative	False Positive	False Negative	True Positive
LeNet	RMSprop	204	14	9	239
LeNet	Adam	211	7	15	233
LeNet	SGD	212	6	13	235

**Table 3:** Validation statistical analysis of the LeNet model.

CNN Architecture	Accuracy	Sensitivity	Specificity	PPV	NPV	FOR	F1-Score
LeNet RMSprop	95.06%	96.37%	93.58%	94.47%	95.77%	4.23%	95.41%
LeNet Adam	95.18%	93.7%	96.79%	96.96%	93.36%	6.63%	95.3%
LeNet SGD	95.92%	94.76%	97.25%	97.51%	94.22%	5.78%	96.11%

**Table 4:** AlexNet confusion matrix (validation).

CNN Architecture	Optimizer	True Negative	False Positive	False Negative	True Positive
AlexNet	RMSprop	206	12	10	238
AlexNet	Adam	205	13	7	241
AlexNet	SGD	212	6	6	242

**Table 5:** Validation statistical analysis of the AlexNet model.

CNN Architecture	Accuracy	Sensitivity	Specificity	PPV	NPV	FOR	F1-Score
AlexNet RMSprop	95.28%	95.97%	94.5%	95.20%	95.37%	4.63%	95.58%
AlexNet Adam	95.71%	97.18%	94.04%	94.88%	96.70%	3.30%	96.02%
AlexNet SGD	97.42%	97.58%	97.25%	97.58%	97.25%	2.75%	97.58%

**Table 6:** VGG16 confusion matrix (validation).

CNN Architecture	Optimizer	True Negative	False Positive	False Negative	True Positive
VGG16	RMSprop	204	14	21	227
VGG16	Adam	203	15	19	229
VGG16	SGD	209	9	21	227

**Table 7:** Validation Statistical Analysis of the VGG16 model.

CNN Architecture	Accuracy	Sensitivity	Specificity	PPV	NPV	FOR	F1-Score
VGG16 RMSprop	92.49%	91.53%	93.58%	94.19%	90.67%	9.33%	92.84%
VGG16 Adam	92.70%	92.34%	93.12%	93.85%	91.44%	8.56%	93.09%
VGG16 SGD	93.56%	91.53%	95.87%	96.19%	90.87%	9.13%	93.80%

**Table 8:** ResNet 50 confusion matrix (validation).

CNN Architecture	Optimizer	True Negative	False Positive	False Negative	True Positive
ResNet 50	RMSprop	207	11	18	230
ResNet 50	Adam	211	7	16	234
ResNet 50	SGD	212	6	11	237

**Table 9:** Validation statistical analysis of the ResNet 50 model.

CNN Architecture	Accuracy	Sensitivity	Specificity	PPV	NPV	FOR	F1-Score
ResNet 50 RMSprop	93.78%	92.74%	94.95%	95.44%	92.0%	8.0%	94.07%
ResNet 50 Adam	95.09%	93.60%	96.79%	97.10%	92.95%	7.04%	95.32%
ResNet 50 SGD	96.35%	95.56%	97.25%	97.53%	95.07%	4.93%	96.54%

**Table 10:** Inception-V1 confusion matrix (validation).

CNN Architecture	Optimizer	True Negative	False Positive	False Negative	True Positive
Inception-V1	RMSprop	206	12	28	220
Inception-V1	Adam	210	8	20	228
Inception-V1	SGD	211	7	16	232

**Table 11:** Validation statistical analysis of the Inception-V1 model.

CNN Architecture	Accuracy	Sensitivity	Specificity	PPV	NPV	FOR	F1-Score
Inception-V1 RMSprop	91.42%	88.71%	94.50%	94.83%	88.03%	11.97%	91.67%
Inception-V1 Adam	93.99%	91.94%	96.33%	96.61%	91.30%	8.70%	94.21%
Inception-V1 SGD	95.06%	93.55%	96.79%	97.07%	92.95%	7.05%	95.28%

**Table 12:** Detection results of AlexNet with the SGD optimizer on the LUNA16 dataset.

		Detection Class	
		Benign	Malignant
**Actual Class**	Benign	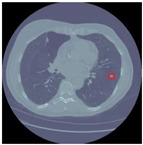	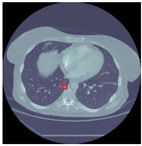
	Malignant	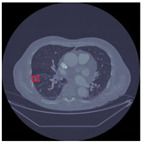	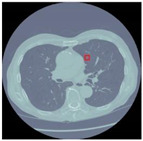

**Table 13:** Validation statistical analysis of five CNN architectures with the SGD optimizer confusion matrix (5-fold cross-validation).

CNN Architecture	Accuracy	Sensitivity	Specificity	PPV	NPV	FOR	F1-Score
LeNet SGD	93.56%	92.74%	94.50%	95.04%	91.96%	8.045%	93.88%
AlexNET SGD	95.73%	95.20%	96.33%	96.75%	94.59%	5.41%	95.97%
VGG16 SGD	93.56%	89.92%	97.71%	97.81%	89.50%	10.50%	93.70%
ResNet-50 SGD	95.28%	96.77%	93.58%	94.49%	96.23%	3.77%	95.62%
Inception-V1 SGD	91.85%	87.1%	97.25%	97.30%	86.89%	13.11%	91.91%

**Table 14:** Comparison of AlexNet with the SGD model with previously published approaches.

Publications	Method	Dataset	Accuracy%	Misclassification Rate
Khehrah et al. [17]	ANN	LIDC-IDRI	92.0%	8.0%
Xie et al. [18]	ANN	LIDC-IDRI	89.62%	10.38%
Naseer et al. [19]	ANN	Private Lung Dataset	96.67%	3.33%
[20]	ANN	Private Lung Dataset	96.67%	3.33%
S. Zhang et al. [22]	LeNet-5 10-fold Cross-Validation	LIDC-IDRI	97.04%	2.96%
Zhao et al. [23]	Hybrid CNN of LeNet and AlexNet	LIDC-IDRI	87.7%	12.3%
Agarwal et al. [25]	AlexNet CNN	Private Lung Dataset	96.0%	4.0%
Polat et al. [26]	Hybrid 3D-CNN RBF-based	LUNA16 Lungs Data Science Bowl	91.81%	8.19%
Al-Yasriy et al. [29]	AlexNet CNN	(IQ-OTH/NCCD) Lung Cancer Dataset	93.548%	6.45%
A. Elnakib et al. [31]	VGG19 architecture and SVM classifier	Early Lung Cancer Action Project (ELCAP) Database	96.25%	3.75%
Nibali et al. [35]	ResNet-18 architecture	LIDC-IDRI	89.90%	10.1%
Zheng et al. [36]	Inception CNN classifier	AIA-INF	88.67%	11.33%
Haibo et al. [37]	DarkNet-53 CNN architecture	LUNA16	73.9%	26.1%
Best Model: AlexNet SGD with 5-fold Cross-Validation	AlexNet with SGD	LUNA16	95.73%	4.27%
Best Model: AlexNet with SGD	AlexNet with SGD	LUNA16	97.42%	2.58%

## Data Availability

The simulation files/data used to support the findings of this study are available from the corresponding author upon request.

## References

[1] Gonzalez T.F. (2007). Handbook of Approximation Algorithms and Metaheuristics.

[2] Christie J.R., Lang P., Zelko L.M., Palma D.A., Abdelrazek M., Mattonen S.A. (2021). Artificial Intelligence in Lung Cancer: Bridging the Gap Between Computational Power and Clinical Decision-Making. Can. Assoc. Radiol. J..

[3] Munir K., Frezza F., Rizzi A. (2021). Deep Learning for Brain Tumor Segmentation. Stud. Comput. Intell..

[4] Rajaraman S., Ganesan P., Antani S. (2022). Deep learning model calibration for improving performance in class-imbalanced medical image classification tasks. PLoS ONE.

[5] Wang S., Yang D.M., Rong R., Zhan X., Fujimoto J., Liu H., Minna J., Wistuba I.I., Xie Y., Xiao G. (2019). Artificial intelligence in lung cancer pathology image analysis. Cancers.

[6] Melekoodappattu J.G., Dhas A.S., Kandathil B.K., Adarsh K.S. (2022). Breast cancer detection in mammogram: Combining modified CNN and texture feature based approach. J. Ambient Intell. Humaniz. Comput..

[7] Liu X., Deng Z., Yang Y. (2019). Recent progress in semantic image segmentation. Artif. Intell. Rev..

[8] Tunali I., Gillies R.J., Schabath M.B. (2021). Application of Radiomics and Artificial Intelligence for Lung Cancer Precision Medicine. Cold Spring Harb. Perspect. Med..

[9] Santos M., Rocha N.P. (2021). Medical Imaging Repository Contributions for Radiation Protection Key Performance Indicators. Procedia Comput. Sci..

[10] Abel M.F., Sutherland D.H., Wenger D.R., Mubarak S.J. (1994). Evaluation of ct scans and 3-D reformatted images for quantitative assessment of the hip. J. Pediatr. Orthop..

[11] Siddiqui S.Y., Abbas S., Khan M.A., Naseer I., Masood T., Khan K.M., Al Ghamdi M.A., Almotiri S.H. (2020). Intelligent decision support system for COVID-19 empowered with deep learning. Comput. Mater. Contin..

[12] Leleu O., Basille D., Auquier M., Clarot C., Hoguet E., Baud M., Lenel S., Milleron B., Berna P., Jounieaux V. (2022). Results of Second Round Lung Cancer Screening by Low-Dose CT scan—French Cohort Study (DEP-KP80). Clin. Lung Cancer.

[13] Bhandary A., Prabhu G.A., Rajinikanth V., Thanaraj K.P., Thanaraj K.P., Satapathy S.C., Robbins D.E., Shasky C., Zhang Y.-D., Tavares J.M.R.S. (2020). Deep-learning framework to detect lung abnormality—A study with chest X-ray and lung CT scan images. Pattern Recognit. Lett..

[14] Gupta P., Shukla A.P. Improving Accuracy of Lung Nodule Classification Using AlexNet Model. Proceedings of the 2021 International Conference on Innovative Computing, Intelligent Communication and Smart Electrical Systems (ICSES).

[15] Shimazaki A., Ueda D., Choppin A., Yamamoto A., Honjo T., Shimahara Y., Miki Y. (2022). Deep learning-based algorithm for lung cancer detection on chest radiographs using the segmentation method. Sci. Rep..

[16] Qi L.-L., Wu B.-T., Tang W., Zhou L.-N., Huang Y., Zhao S.-J., Li M., Zhang L., Feng S.-C., Hou D.-H. (2020). Long-term follow-up of persistent pulmonary pure ground-glass nodules with deep learning–assisted nodule segmentation. Eur. Radiol..

[17] Khehrah N., Farid M.S., Bilal S., Khan M.H. (2020). Lung Nodule Detection in CT Images Using Statistical and Shape-Based Features. J. Imaging.

[18] Xie Y., Zhang J., Xia Y., Fulham M., Zhang Y. (2018). Fusing texture, shape and deep model-learned information at decision level for automated classification of lung nodules on chest CT. Inf. Fusion.

[19] Nasser I.M., Abu-Naser S.S. (2019). Lung Cancer Detection Using Artificial Neural Network. Int. J. Eng. Inf. Syst..

[20] Miah M.B.A., Yousuf M.A. Detection of lung cancer from CT image using image processing and neural network. Proceedings of the 2015 International Conference on Electrical Engineering and Information Communication Technology (ICEEICT).

[21] LeCun Y., Boser B., Denker J., Henderson D., Howard R., Hubbard W., Jackel L. (1989). Handwritten Digit Recognition with a Back-Propagation Network. Adv. Neural Inf. Process. Syst..

[22] Zhang S., Sun F., Wang N., Zhang C., Yu Q., Zhang M., Babyn P., Zhong H. (2019). Computer-Aided Diagnosis (CAD) of Pulmonary Nodule of Thoracic CT Image Using Transfer Learning. J. Digit. Imaging.

[23] Zhao X., Liu L., Qi S., Teng Y., Li J., Qian W. (2018). Agile convolutional neural network for pulmonary nodule classification using CT images. Int. J. Comput. Assist. Radiol. Surg..

[24] Krizhevsky B.A., Sutskever I., Hinton G.E. (2012). Imagenet classification with deep convolutional neural networks. Adv. Neural Inf. Processing Syst..

[25] Agarwal A., Patni K., Rajeswari D. Lung Cancer Detection and Classification Based on Alexnet CNN. Proceedings of the 2021 6th International Conference on Communication and Electronics Systems (ICCES).

[26] Polat H., Mehr H.D. (2019). Classification of pulmonary CT images by using hybrid 3D-deep convolutional neural network architecture. Appl. Sci..

[27] Rao P., Fereira N.A., Srinivasan R. Convolutional neural networks for lung cancer screening in computed tomography (CT) scans. Proceedings of the 2016 2nd International Conference on Contemporary Computing and Informatics (IC3I).

[28] Lin C.J., Li Y.C. (2020). Lung nodule classification using taguchi-based convolutional neural networks for computer tomography images. Electronics.

[29] Al-Yasriy H.F., Al-Husieny M.S., Mohsen F.Y., Khalil E.A., Hassan Z.S. (2020). Diagnosis of Lung Cancer Based on CT Scans Using CNN. IOP Conf. Ser. Mater. Sci. Eng..

[30] Simonyan K., Zisserman A. (2015). Very deep convolutional networks for large-scale image recognition. arXiv.

[31] Elnakib A., Amer H.M., Abou-Chadi F.E.Z. (2020). Early lung cancer detection using deep learning optimization. Int. J. Online Biomed. Eng..

[32] Geng L., Zhang S., Tong J., Xiao Z. (2019). Lung segmentation method with dilated convolution based on VGG-16 network. Comput. Assist. Surg..

[33] Pang S., Meng F., Wang X., Wang J., Song T., Wang X., Cheng X. (2020). VGG16-T: A novel deep convolutional neural network with boosting to identify pathological type of lung cancer in early stage by ct images. Int. J. Comput. Intell. Syst..

[34] Sajja T.K., Devarapalli R.M., Kalluri H.K. (2019). Lung cancer detection based on CT scan images by using deep transfer learning. Trait. Signal.

[35] Nibali A., He Z., Wollersheim D. (2017). Pulmonary nodule classification with deep residual networks. Int. J. Comput. Assist. Radiol. Surg..

[36] Zheng G., Han G., Soomro N.Q. (2019). An Inception Module CNN Classifiers Fusion Method on Pulmonary Nodule Diagnosis by Signs. Tsinghua Sci. Technol..

[37] Haibo L., Shanli T., Shuang S., Haoran L. An improved yolov3 algorithm for pulmonary nodule detection. Proceedings of the 2021 IEEE 4th Advanced Information Management, Communicates, Electronic and Automation Control Conference (IMCEC).

[38] Zhang X., Lee V.C.S., Rong J., Liu F., Kong H. (2022). Multi-channel convolutional neural network architectures for thyroid cancer detection. PLoS ONE.

[39] Leo M., Cacagnì P., Signore L., Benincasa G., Laukkanen M.O., Distante C. (2022). Improving Colon Carcinoma Grading by Advanced CNN Models. International Conference on Image Analysis and Processing.

[40] Siddiqui S.Y., Naseer I., Khan M.A., Mushtaq M.F., Naqvi R.A., Hussain D., Haider A. (2021). Intelligent breast cancer prediction empowered with fusion and deep learning. Comput. Mater. Contin..

[41] Siddiqui S.Y., Haider A., Ghazal T.M., Khan M.A., Naseer I., Abbas S., Rahman M., Khan J.A., Ahmad M., Hasan M.K. (2021). IoMT Cloud-Based Intelligent Prediction of Breast Cancer Stages Empowered with Deep Learning. IEEE Access.

[42] Carcagnì P., Leo M., Celeste G., Distante C., Cuna A. A systematic investigation on deep architectures for automatic skin lesions classification. Proceedings of the 2020 25th International Conference on Pattern Recognition (ICPR).

[43] Zhang N., Cai Y.X., Wang Y.Y., Tian Y.T., Wang X.L., Badami B. (2020). Skin cancer diagnosis based on optimized convolutional neural network. Artif. Intell. Med..

[44] Setio A.A.A., Traverso A., de Bel T., Berens M.S.N., van den Bogaard C., Cerello P., Chen H., Dou Q., Fantacci M.E., Geurts B. (2017). Validation, comparison, and combination of algorithms for automatic detection of pulmonary nodules in computed tomography images: The LUNA16 challenge. Med. Image Anal..

[45] Kazerooni E.A., Austin J.H.M., Black W.C., Dyer D.S., Hazelton T.R., Leung A.N., McNitt-Gray M.F., Munden R.F., Pipavath S. (2014). ACR-STR practice parameter for the performance and reporting of lung cancer screening thoracic computed tomography (CT): 2014 (Resolution 4). J. Thorac. Imaging.

[46] Li Y., Tang Y. (2022). Design on Intelligent Feature Graphics Based on Convolution Operation. Mathematics.

[47] Han Y., Li J., Lou X., Fan C., Geng Z. (2022). Energy saving of buildings for reducing carbon dioxide emissions using novel dendrite net integrated adaptive mean square gradient. Appl. Energy.

[48] Mao Q., Zhao S., Ren L., Li Z., Tong D., Yuan X., Li H. (2021). Intelligent immune clonal optimization algorithm for pulmonary nodule classification. Math. Biosci. Eng..

[49] Gao Y., Song F., Zhang P., Liu J., Cui J., Ma Y., Zhang G., Luo J. (2021). Improving the Subtype Classification of Non-small Cell Lung Cancer by Elastic Deformation Based Machine Learning. J. Digit. Imaging.

[50] Lai K.D., Nguyen T.T., Le T.H. (2021). Detection of lung nodules on ct images based on the convolutional neural network with attention mechanism. Ann. Emerg. Technol. Comput..

[51] Silva F., Pereira T., Neves I., Morgado J., Freitas C., Malafaia M., Sousa J., Fonseca J., Negrao E., de Lima B.F. (2022). Towards Machine Learning-Aided Lung Cancer Clinical Routines: Approaches and Open Challenges. J. Pers. Med..

[52] Pradhan K., Chawla P. (2020). Medical Internet of things using machine learning algorithms for lung cancer detection. J. Manag. Anal..

[53] Bansal G., Chamola V., Narang P., Kumar S., Raman S. (2020). Deep3DScan: Deep residual network and morphological descriptor based framework for lung cancer classification and 3D segmentation. IET Image Process..

